# Elective Localization and Focal Infection from Oral Sepsis*These extracts are taken from a paper which is printed in full in the November *Journal of the N. D. A*.

**Published:** 1919-12

**Authors:** E. C. Rosenow


### ELECTIVE LOCALIZATION AND FOCAL INFECTION FROM ORAL SEPSIS*

BY E. C. ROSENOW, M.D.

That foci of infection, often insignificant and symptomless in themselves, are a cause of systemic disease is indicated by many facts. Clinical studies have shown that focal infections are present in demonstrable form in a high percentage of the sick, including children. Thorough removal of foci of infection is followed by improvement in the general health and frequently by improvement or cure of distant local and systemic diseases, provided the changes are not too far advanced. Indeed, improvement has occurred so often that many physicians have come to regard these observations as proof of etiologic relationship, and if improvement does not follow the removal of a given focus it is considered presumptive evidence either that the particular operation was not properly performed or that other foci exist. Exacerbations of systemic conditions immediately following the removal of certain foci of infection further suggests causal relationship. The idea of an etiologic relationship is not new, and relief of systemic conditions following the removal of foci of infection is mentioned in the older medical literature.

Rush in 1801, cited by Duke, after mentioning a number of striking cures which followed extraction of diseased teeth made the following statement: ''I have been made happy by discovering that I have only added to the observations of other physicians, in pointing out a connection between the extraction of decayed and diseased teeth and the cure of general diseases.'' Miller, in 1889 demonstrated that infection of the mouth may cause constitutional disease. Pyorrhea is mentioned as a causative factor in pathologic

* These extracts are taken from a paper which is printed in full in the November *Journal of the N. D. A.*

condition of the joints and tonsilitis as frequently accompanying rheumatic fever. A. D. Black emphasizes the importance of dental lesions in ocular disease, and in a review of the older medical literature, gives numerous instances indicating causal relationship between dental lesions, particularly in the form of alveolar abscess at the root apex, and pus pockets along the side of the root, to diseases of the eye. The relationship between ill health and infections of the mouth was thought in earlier years to be due chiefly to the swallowing of pus and of putrid material.

Notwithstanding the repeated suggestions of etiologic relationship, the medical and dental professions, as a whole, remained indifferent until Billings, in *Journal A. M. A.*, 1914, and his co-workers made their extensive clinical observations and correlated experimental studies in animals, demonstrating the importance of septic foci, even when small, as a source of chronic infections conveyed by the blood stream. The broader conception of this interrelationship, well expressed by the term "focal infection," may therefore be regarded as having had its origin in recent years. The experimental studies included dental foci as well as localized infections in other parts of the body. As early as 1908, Rosenow, in the *Journal of Infectious Diseases*, 1909, p. 245, endocarditis was produced by intravenous injection of the respective strains from a case each of staphylococus and streptococcus viridans endocarditis, which were clearly the result of dental infections. The combined clinical and experimental studies of Hartzell, Henrici, Gilmer and Moody, emphasized the importance of dental foci as sources of systemic infections. For a detailed consideration of results of numerous clinical and roentgenologic studies, and an exhaustive bibliography, Duke's excellent monograph on this subject should be consulted.

### METASTATIC INFECTION

Many facts suggest that dental infections are often metastatic from foci elsewhere than in the dental area.

The observation which I have made, that bacteria isolated from the tissues in metastatic lesions show a greater affinity for that tissue than those from the primary focus, seems to indicate that the repeated occurrence of the same type of lesion, such as pulpitis in a given case, may be the result of a blood-borne infection from one pulp to another, as well as from infection from a focus in the tonsil or elsewhere. The rather common occurrence of pulpitis during or following certain epidemics of acute respiratory diseases is in harmony with this idea. That infection of the pulp may be metastatic is shown by the fact that it occurs in sound teeth, that apical abscesses are sometimes found at the roots of otherwise normal teeth whose pulp chambers have not been perforated either by treatment or decay, and that infection is occasionally found in unerupted teeth. Moreover, direct experimental evidence that this may occur has been obtained. In a previous communication, I have shown the affinity for dental pulps and dental nerves in animals of a streptococcus isolated from the foul pulp of the tooth of a person in whom infection of a number of pulps subsequently occurred.

### DANGER OF EXTENSIVE EXTRACTIONS OF DISEASED TEETH

The fact is now well known that acute exacerbations in symptoms follow the surgical removal of certain foci of infection in chronic diseases, especially those about the teeth. This is particularly likely to occur in persons with chronic disorders, who appear to be hypersensitive. The danger from the removal of too many foci at one time, in these cases, was noted some years ago when a fatal exacerbation following the removal of and curettement of a number of abscessed teeth occurred in a patient suffering from chronic arthritis.

On February 6, 1913, tonsillectomy was followed by slight increase in temperature for a day or two. On February 16th, a number of teeth were extracted, followed on the next day by fever from 102 to 105 F. for nearly three weeks, associated with pericarditis, pleurisy with effusion, bronchopneumonia, exacerbation of the joint sensitiveness, and successive crops of arythematous nodes of the skin chiefly over the forearms and legs, acute dilatation with acute multiple ulceration of the stomach shortly before death, February 28th.''—Dr. Rosenow, in *Journal N. D. A.*

The sockets were curetted immediately after extraction. The importance of the gradual removal of foci, particularly in the dental area in chronic diseases, has since been emphasized especially by Hartzell, Thoma and many others. The exacerbations may be due to the absorption of toxic material to which these patients are hypersensitive, to a lighting up of a more or less dormant infection in metastatic lesions, or to a localization of bacteria that gain entrance through the traumatized area from which the diseased tissue may have been only partly removed. That such localization occurs in some instances is indicated by the results obtained in the case quoted above, and by the fact that the bacteria contained in the material expressed from tonsils or contained in extirpated tonsils, in some persons who develop temporary exacerbations, showed elective localizing power in animals. Thus, a patient with simple thoracic herpes zoster developed a gangrenous herpes zoster following the expression of pus from the tonsils containing bacteria that produced herpes in animals.

The results reported heretofore on elective localization of bacteria from foci of infection have been verified and extended. The findings warrant the conclusion that chronic foci of infection about the teeth are potentially or actually detrimental to the health of the persons who harbor them. The lesions which are more or less enclosed, and which drain only into the circulation, are probably the most dangerous and should be regarded as veritable experiments which alone or in connection with predisposing factors will sooner or later break down the resistance of the patient and produce disease. The harm from oral sepsis, according to the experiments with emulsions from the infected tonsils and with the bacteria in the pus from tonsils, may be due to absorption of poisonous bacteria products and to the living bacteria themselves. The place of localization of the bacteria, aside from the influence of a lowered resistance, local or general, from injury, fatigue, strain, improper food, bad hygiene, disease, heredity, etc., will depend largely on the peculiar infective, of the peculiar poison-producing power of the bacteria at hand.

### CLINICAL FINDINGS

Let us consider the frequency of probably the most dangerous form of dental sepsis, pulpless teeth and blind abscesses. Howe, reports the presence of 40,000 abscessed teeth in an examination of 50,000 children at the Forsythe Dental Infirmary, and points out that abscessed teeth must be of little importance as a source of infection since arthritis deformans (a disease which rarely occurs in childhood) was not observed. It is to be regretted that no information is given with regard to the condition of these children at the time of the examination, for example, the number who later suffered from malnutrition, hypertrophied and infected tonsils, and adenoids leading to malformation of the jaw, defective teeth, and to deficient mental and physical development, and the number who had appendicitis, endocarditis and so-called ''idiopathic'' infections, fatal or otherwise, in which the teeth were not even suspected as being a possible source of these infections. Langstroth, in his studies of cases at the University of California Hospital, found chronic focal infections in 84 per cent. of ulcer patients, in 66 per cent. of subacute cases of arthritis, in 73 per cent. of the chronic cases of arthritis, and in 100 per cent. of the gall-bladder cases. The acute and subacute cases responded well after removal of the foci even to the point of absolute care. In many of the chronic cases, the patients had less pain and no further progression of the disease. Duke, in tabulating 1000 medical cases in which the patients suffered from some from of chronic disease, found a marked degree of oral sepsis in 66 per cent. Thoma, in a similar group of cases at the Robert B. Brigham Hospital in Boston, found alveolar abscess in 88 per cent. Irons, found alveolar abscess in 44 per cent. in a series of 124 patients with miscellaneous diseases. Abscesses were present in 76 per cent. of the arthritis group and in 47 per cent. of the nephritis group. A. D. Black, found that the peridental infections, without reference to complaint, were 56 per cent. in persons under 25 years of age, 72 per cent. in persons between 25 and 30, 87 per cent. in persons between 30 and 40, 89 per cent. in persons between 40 and 50, and 100 per cent. in persons more than 50. Many of these infections are found in teeth from which the pulp has been removed artificially and the canals improperly filled as is shown by Ulrich, who found that 68 per cent. of all artificially devitalized teeth showing apical abscesses, and that of 1350 so-called dead teeth examined 83 per cent. were abscessed. The number of persons suffering from diseases directly attributable to these infections as well as from non-related conditions which have been cured or benefited by elimination of foci of infection in the various branches of medicine is so large as to be quite sufficient to prove the general truth of the idea of causal relationship.

THERAPEUTIC SUGGESTIONS

The opportunity of the dental profession for co-operation with the various branches of medicine along these lines needs no emphasis. The prevention of oral sepsis in the future with a view to lessening the incidence of systemic disease should henceforth take precedence in dental practice, over the preservation of the teeth almost wholly for mechanical or cosmetic purposes as has been so largely the case in the past. Every effort should be made for the prevention of dental infections and for the correction of those already present. Preventive measures should begin in childhood with a view to obtain perfect development of the teeth and oral cavity, and thus prevent various defects which would later lead to sepsis. This calls for the co-operation of dentist, pediatrist and throat specialist.

The principles underlying various procedures for the prevention and cure of infections of the gums and enveloping membranes about the roots of teeth may be regarded as fairly well understood and effectively applied by many. It should be emphasized, however, that the chief harm from these conditions comes from the absorption of the bacteria and their products into the lymph stream or blood, especially if drainage is inadequate, not from swallowing the infectious material, and that the infections predispose to embolic infections within and without the dental field. The correction of pyorrhea and allied conditions is, therefore, of great importance.

Infections of the dental pulp, pulpless teeth and apical abscesses are theoretically the most dangerous of the various forms of dental foci. They are usually free from symptoms and hence unsuspected. They are situated in osseous tissue which allows no expansion. They lack drainage other than into the circulation and are exposed to pressure transmitted by the teeth during mastication. They remain active and do not heal for a period of years, and the bacteria, as shown in this study, are not encapsulated as is usually assumed, but are found in areas of active inflammatory reaction where new blood vessels form and afford ample drainage into the circulation.

On the basis of these facts, there can be no doubt that the wholesale devitalization of teeth, often for trivial reasons, and the filling of infected root-canals without due regard to asepsis, as practiced in the past, results in the formation of numerous apical infections, and no doubt in much ill health. The instances of cure or improvement in systemic diseases directly attributable to these infections and in non-related conditions are so numerous that the procedure as practiced heretofore should be regarded as a veritable experiment.

It is a well-known fact in bone surgery that no amount of antiseptic treatment will cure an osteomyelitis unless dead tissue is removed and dead spaces are eliminated; if this is done, healing occurs promptly without antiseptic treatment. In the filling of root-canals the removal of every particle of pulp tissue is recognized as a prerequisite for successfully preventing subsequent infection. Is there any reason to believe that if the small amount of dead albuminous matter in divergent or tortuous canals leads to reinfection, that the larger amount in the apical region would not likewise become infected even though completely sterilized by ionization or other similar treatment? Hence, it is doubtful whether any form of medication through the root-canal, which would be applicable in routine practice, can be relied on successfully to sterilize the infected areas about abscessed teeth, and to prevent the areas from becoming reinfected. The fact that acute infections of the jaw occur not infrequently following these attempts is a further obstacle to the success of this method. Apicoectomy, while no doubt successful in removing the infection in the jaw in some instances, is applicable in only a small number of cases, and as a rule should not be attempted in persons who are ill from secondary systemic conditions. The removal of infected pulpless teeth together with the infected peridental tissues therefore, seems to be the safest and surest means available at present for the cure of these conditions. The many ingenious devices applicable to vital teeth will do much in making useful masticating surfaces and agreeable cosmetic effects.

It is becoming more and more apparent that the lack of improvement in systemic disease following the extraction of one or more infected teeth, barring other foci, may be due to the fact that the peridental infection was left or was only partially removed, and that the occurrence of acute exacerbations following extraction and curettement is commonly due to this cause. Persons who have had all their teeth extracted may still harbor localized areas of infection in the jaws. Simple extraction is not sufficient. The importance of eliminating dead spaces in curing infections of bone in other parts of the body—a lesson learned during the war—lends support to the idea of the "surgical removal" of infected teeth. Removal would seem to be the method of choice in cases of extensive apical infections. If a person is perfectly well, has harbored for some years one or more devitalized teeth in which the X-ray findings are negative, there would seem to be no good reason for extraction. If, on the other hand, the person is suffering from arthritis, a heart or kidney affection, or some other form of disease for which other causes can not be found, such teeth should be removed. Owing to the reparative power of the cementum, it would seem possible to devitalize teeth safely whose pulps are sterile and whose canals may be properly filled, provided the operation is done in an aseptic manner. This should be done after the removal of other sources of infection, and only in teeth of vital importance for restorative needs. The somewhat lowered resistance to infection of the peridental tissues about non-vital, sterile teeth, may be more than counterbalanced by the removal of the pulp, since most infections of otherwise sound teeth no doubt occur through this organ. In case of beginning infection of the pulp from decay which has not yet extended into the peridental tissues, attempts at sterilization, and if necessary, removal of the pulp and filling the canal may be justified in some instances, but not until cultures have proved that the tissues are free from living bacteria, and only if the responsibility is shared conjointly by patient and operator.

In the manner of eliminating foci of infection in the mouth and throat, the infections about teeth should, as a rule, be corrected first. Tonsillectomy as now so commonly practiced before the condition of the teeth has been corrected is illogical. The lymphatics of the mouth and jaws drain into the tonsils. Some infections of tonsils improve or even disappear following the extraction of infected teeth.

The unnecessary sacrifice of vital teeth should be condemned. A patient who is suffering from a serious disease of focal origin, after due consideration of other factors for or against extraction of infected teeth, should be given the benefit of the doubt even when the evidence is not conclusive that the responsible focus is contained therein. Extraction will do no harm although inconvenient at the time, while non-extraction may result seriously. Not too much should be expected from the removal of a focus, especially in chronic conditions, because a similar condition may be present in inaccessible foci and in others too small to be detected. Moreover, recovery may be made difficult by local tissue sensitivity or peculiar mechanical conditions, and living bacteria in a metastatic lesion may continue the process independently of the focal source.

The administration in guarded dosage of a properly prepared vaccine from a focus following its removal may be an important aid in overcoming the metastatic condition. But this or other forms of specific therapy can not take the place of the eradication of the focus. It need hardly be pointed out that the prevention and cure of dental and other foci of infection is only a part of the problem. The conception of the problem of focal infection must not be too mechanical, for the invasive power of the bacteria is extremely important.

Thus in the case of pulpitis, dental neuritis and myositis in which elective localization of the streptococci from the pulp of the tooth and from the muscle was demonstrated, the streptococcus having the same invasive power was found also in the nose, pharynx and stool.

The elimination of visible foci would not eliminate all systemic infections, for as the invasive power of the bacteria increases, the need for forced entrance, as occurs from a focus, becomes less marked, and thus the bacteria may gain entrance through the unabraded mucous membranes, as in the acute infectious diseases. But even here, the broken continuity from localized infections predisposes to these diseases. The importance of hygiene, of a properly balanced diet, and of the general health, and hence the co-operation between the various branches of medicine, surgery and dentistry, need hardly be emphasized.

A careful study of the clinical and experimental data now available seems to show conclusively that a sane and comprehensive effort toward the prevention and cure of septic foci in the dental and other areas will result in the alleviation of human suffering; in a better preservation of the tissues in old age, in a longer average duration of life; in increased mental and physical efficiency; in the prevention and cure of acute and chronic diseases, and through the laws of heredity, make for a sturdier race.

